# Keystone Veterinary Medical Association

**Published:** 1895-12

**Authors:** W. L. Rhoads

**Affiliations:** Secretary


### KEYSTONE VETERINARY MEDICAL ASSOCIATION.

THE regular monthly meeting was called to order at the new quarters, northwest corner of Broad and Filbert Streets, Philadelphia, by President J. R. Hart, at 8 P.M., Tuesday evening, November 12th, with the following present: T. B. Rayner, J. B. Rayner, W. S. Kooker, J. R. Hart, C. Lintz, F. Bridge, J. T. McAnulty, W. H. Hoskins, and W. L. Rhoads.

Dr. Hoskins, as Chairman of the Legislative Committee, reported that the State Board of Veterinary Medical Examiners were to hold their initial meeting at Harrisburg on the 13th, at 12 o'clock, for organization, the Governor having made the following appointments: Drs. W. H. Hoskins and S. J. J. Harger, of Philadelphia, for three years; Drs. J. C. McNeil, of Pittsburg, and Harry Walters, of Wilkesbarre, for two years; and Dr. J. W. Sallade, of Pottsville, for one year. The question of the Board accepting the diploma of any school as sufficient without an examination by the Board was broached, and very vigorous protests were made against the Board thus shirking their duty and responsibility, and evading the law. It was moved and unanimously adopted as the sense of this Association that all who enter the practice of veterinary science in Pennsylvania must be examined by the Board and pass all requirements.

Dr. Hoskins asked for information regarding true hemorrhoids in the horse, as he thought such a condition impossible on account of anatomical structure and position. He stated his inability to find any record of such cases in veterinary literature of the last fourteen years. This question was spoken upon at some length by Drs. J. B. Rayner, W. S. Kooker, F. Bridge, T. B. Rayner, and C. Lintz—all spoke of a hemorrhoidal condition occasionally found, but all agreed they had never seen true hemorrhoids in our equine friend.

Dr. Kooker mentioned an affection among some horses under his care.

They could not be induced to eat, and became dull and showed diarrhœa when driven; otherwise they were perfectly normal. Dr. Hoskins mentioned a case of osteoporosis in a pony two and one-half years old, said trouble coming when he seemed to be in the best possible physical condition and high spirits.

Meeting adjourned to reconvene December 10th.

W. L. RHOADS,
Secretary.

